# Accommodation and Binocular Vision in Children with Myopic Anisometropia

**DOI:** 10.1155/2024/6525136

**Published:** 2024-01-16

**Authors:** Chu-chu Zhuang, Ling Zhang, Shan-shan Pan, Yi-ning Wang, Jian-xin Guo

**Affiliations:** ^1^The First Medicine College, Xuzhou Medical University, Xuzhou 221004, China; ^2^Department of Ophthalmology, Affiliated Hospital of Xuzhou Medical University, Xuzhou 221004, China

## Abstract

**Purpose:**

To assess the differences in accommodation and binocular vision in children with myopic anisometropia and determine the correlation with anisometropia.

**Method:**

A total of 110 patients with myopia aged 8–15 years were recruited from June 2021 to February 2022 from the Affiliated Hospital of Xuzhou Medical University. Based on the interocular differences of spherical equivalent refraction, patients were divided into the isometropia (35 children), low anisometropia (LA group, 42 children), and high anisometropia (HA group, 33 children). The variables assessed were refraction, heterophoria, amplitude of accommodation (AMP), accommodative response (AR), gradient AC/A, positive and negative relative accommodation (PRA/NRA), and near stereopsis in the three groups. Pearson's correlation coefficient tests were used to investigate the possible association between each parameter and interocular differences (IODs).

**Results:**

Among 110 subjects, there were 49 males and 61 females with a mean age of 11.39 ± 2.28 years. Compared with those in the isometropia group, AMP was lower and near stereopsis was higher in the LA group, and the distance and near heterophoria, PRA, AR, and near stereopsis were higher, and PRA, AMP, and gradient AC/A were lower in the HA group (all *P* < 0.05). Compared with those in the LA group, the near stereopsis, AR, and the near stereopsis were higher in the HA group, and the gradient AC/A was lower (all *P* < 0.05). However, no significant differences existed in the negative relative accommodation (*P* > 0.05). The distance and near heterophoria, AR, AMP, and near stereopsis were observed to be correlated with IODs, respectively (*r* = −0.259, *p* = 0.006; *r* = −0.201, *p* = 0.036; *r* = 0.306, *p* = 0.001; *r* = −0.315, *p* = 0.001; *r* = 0.535, *p* < 0.001).

**Conclusion:**

Our results suggested that with the increase of anisometropia, distance and near heterophoria, AR, AMP, and near stereopsis had a tendency to get worse in children with myopic anisometropia.

## 1. Introduction

Myopic anisometropia is defined as the threshold interocular difference of 1 diopter (D) or more, generally owing to an asymmetry in axial lengths [1]. Patients with anisometropia often develop diplopia, aniseikonia, decreased stereopsis, and asthenopia owing to the differences in binocular refractive conditions. When the difference in spherical equivalent refractive error is 2.50 D or more, these symptoms generally become apparent [2, 3]. The prevalence of myopic anisometropia was found to be 18.7% in an epidemiological study of 9832 adults with myopia [4], while it increased from 5% in childhood to 22.6% in adulthood in a 23-year follow-up [5]. The development of myopic anisometropia is associated with near-work and outdoor activities [6]. Additionally, with the spread of the coronavirus disease pandemic, students studied online using digital screen devices instead of outdoor activities. Therefore, further studies should be conducted, including eyes with myopic anisometropia and binocular vision, for assessing the adverse effects of interocular differences on binocular vision.

Previous studies on accommodation and myopic anisometropia primarily assessed the asymmetry in axial lengths and anatomical characteristics between the eyes, and most binocular vision have focused on children with myopia [7, 8]. However, there are limited reports on accommodation and binocular vision for patients with myopic anisometropia.

Only a few studies have focused on the specific binocular vision and differences in accommodation between isometropia and anisometropia. Patients with anisometropia are observed to have binocular visual dysfunction challenges such as unequal imaging size between the eyes and contradiction in accommodation and convergence. These visual dysfunction challenges may cause symptoms, including blurred vision, difficulty in focusing, and headaches [9]. Therefore, our study aimed to systematically analyze the differences in accommodation and binocular vision in children with myopic anisometropia and assess the correlation with anisometropia.

## 2. Patients and Methods

### 2.1. Subjects

**Figure 1:**
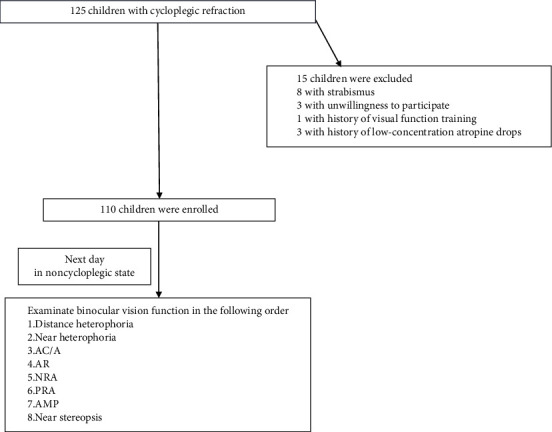
Study flowchart.

A total of 110 children aged 8–15 years were recruited from June 2021 to February 2022 from the Affiliated Hospital of Xuzhou Medical University, China. Based on the interocular differences (IODs) of spherical equivalent refraction, eligible participants were enrolled in three groups. Patients in the low anisometropia group had IODs ≥1.00 D and ≤2.50 D. Those in the high anisometropia group had IODs ≥2.50 D. The isometropia group had IODs ≤1.00 D. This study was approved by the Committee of Research Ethics of the Affiliated Hospital of Xuzhou Medical University (ID: XYFY2022-KL017-01). The examinations and data collection procedures followed the tenets of the Declaration of Helsinki. The written informed consent form was signed by all participants after an explanation of the nature and possible consequences of the study. Record the patient's age, gender, and the time that myopia was first found to be the cause of their blurry vision. Then, participants who met the inclusion criteria were administered 1% tropicamide drops three times at an interval of 5 minutes, and refraction was measured after cycloplegia was noted. The result was converted to SER, which ranged from −6.00 D to −0.50 D. The best-corrected visual acuity (BCVA) was ≤0.1 logarithm of the minimum angle of resolution in either eye. Then, the next day after the ciliary muscle was restored, subjective refraction was performed and binocular vision was measured. The exclusion criteria were as follows: any systemic or eye disease, strabismus, evidence of keratoconus, history of eye surgery, and unwillingness to participate. Moreover, any patients who underwent visual function training or were administered low-concentration atropine drops or orthokeratology within 3 months were also excluded (Figure 1).

### 2.2. Measurements

The following measurements for binocular vision and accommodation were required: heterophoria, the amplitude of accommodation (AMP), accommodative response (AR), gradient AC/A, positive and negative relative accommodation, and near stereoacuity. The following procedures at a distance were performed first.

1. The distance and near horizontal heterophoria (using Von Graefe technique)
2. AMP (minus lens method, the average age-expected AMP based on Hofstetter's formula: 18.5 D − 0.3 *∗* age, then calculated the difference between the measured and the average age-expected values)
3. AR (fused cross cylinder with a Maltese cross target at 40 cm [10])
4. The gradient AC/A (after the first Von Graefe technique, adding −1.00 D to the prescription of distance correction, and comparing the heterophoria twice [11])
5. The positive and negative relation accommodation (PRA/NRA: measured with minus/plus lenses to get the maximum relaxation/stimulate accommodation while maintaining clear, single binocular vision)
6. The near stereoacuity (the titmus stereopsis test [12], Stereo Fly Test Stereo Optical Co. Inc. Stereopsis was converted to log values from 1.30 to 2.90 for analysis)

### 2.3. Statistical Analysis

Statistical analyses were performed using SPSS 26.0. The measurement data were determined using the Shapiro–Wilk test, and data conforming to normal distribution were expressed as the means ± standard deviation, otherwise were expressed as *M* (P25,P75). A one-way analysis of variance with LSD-*t* post hoc test was performed for normally distributed data. The Kruskal–Wallis *H* with Bonferroni post hoc test was used for non-normally distributed data. To investigate the possible association between anisometropia and each parameter of binocular vision, Pearson's correlation coefficient tests were performed. *P* < 0.05 was considered statistically significant.

## 3. Results

A total of 110 eligible subjects (49 males, 61 females) were enrolled. The mean age was 11.39 ± 2.23 years. The mean IOD was 1.71 ± 0.83 D. The demographics of each group are shown in Table 1. The high anisometropia (HA), low anisometropia (LA), and isometropia groups comprised 33, 42, and 35 patients, respectively. No significant difference was found in age or sex among the three groups (all *P* > 0.05). However, significant differences were found in the time of myopia onset and IOD of spherical equivalent refraction (*P* < 0.05).

As shown in Table 2, significant differences were found in heterophoria, PRA, gradient AC/A, AMP, AR, and near stereopsis among the three groups (all *P* < 0.05). However, no significant difference was found in NRA among the three groups (*P* > 0.05). The results of the pairwise comparison depicted that near heterophoria and gradient AC/A in the HA group were lower than those in the LA group, the AR and near stereopsis in the HA group were higher than those in the LA group, and the differences were significant (all *P* < 0.05).

**Figure 2:**
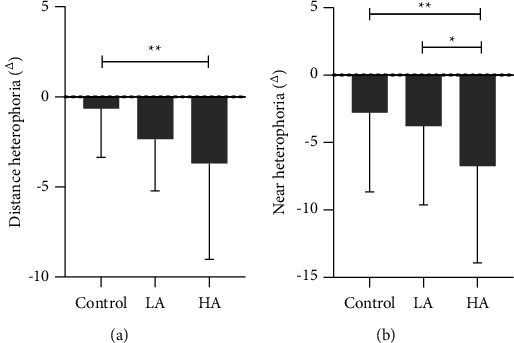
Comparison of heterophoria among three groups.

The heterophoria, AMP, and the gradient AC/A in the HA group were lower than those in the control group. However, PRA, AR, and near stereopsis were higher. In the LA group, AMP was lower; however, the near stereopsis was higher, and the difference was significant (all *P* < 0.05, Table 2, Figure 2).

Our study demonstrated that anisometropia was positively correlated with age, AR, and near stereopsis (*r* = 0.304, *P* = 0.001; *r* = 0.306, *P* = 0.001; *r* = 0.535, *P* < 0.001), and negatively correlated with AMP and distance and near heterophoria (*r* = −0.315, *P* = 0.001; *r* = −0.259, *P* = 0.006; *r* = −0.201, *P* = 0.036). Other parameters were found not correlated with anisometropia (all *P* > 0.05, Table 3).

## 4. Discussion

The potential ocular deviation that can be compensated by the binocular fusion mechanism is referred to as heterophoria; however, when the fusion is insufficient to compensate, and the eyes are in intermittent or constant ocular deviation, it is known as strabismus [13]. This study found that the HA group showed more exophoria and different degrees of exophoria in distant and near horizontal eye positions. We speculated that this finding might be owing to different retinal imaging sizes in patients with anisometropia. Exophoria develops when the fusion ability of the human brain's visual center cannot compensate for the difference in retinal imaging, with difficulty in binocular fusion. In addition, to ensure normal binocular single vision, patients should use more fusion dispersion to compensate for the regulatory set abnormalities, resulting in a decrease in fusion reserve and exophoria.

Our study found that the absolute PRA value in the HA group was significantly higher than that in the control group. Liu and Zhang reported a higher PRA in children with myopic anisometropia than that in the control group, which is consistent with our results [14]. The maximum accommodation that can be relaxed when the eyes are fixed on a close target is referred to as PRA. A lower PRA means that the patient's abnormal binocular visual symptoms, such as eye fatigue are obvious, with insufficient relative accommodation reserve. Some authors believe that this is one of the reasons for the myopia progression [15].

This study found a lower AMP associated with anisometropia, consistent with previous finding [16]. Our study found that AMP decreased with anisometropia, and the gap from the average AMP significantly increased, according to the average age-expected amplitude of accommodation (18.5 − 0.3 *∗* age). Recently, the nasal scleral surface was reported to change significantly in the state of accommodation and convergence using the eye surface profiler instrument [17]. Significant changes in scleral structure, extracellular matrix, and biomechanical properties were associated with myopia development [18, 19]. This suggests that patients with anisometropia may have insufficient accommodation reserve and have to accommodate more than those with emmetropia when looking at the same near object. This results in changes in scleral morphology, which in turn stimulates the progression of myopia. Some scholars speculate that defocusing owing to insufficient accommodation is a stimulating factor that causes myopia progression in axial growth [20]; however, no direct evidence can support it. Therefore, further longitudinal studies are required to explore the linkage between accommodation and anisometropia.

Previous studies have demonstrated that patients with myopia display unusually larger accommodative lags than those who remain emmetropic [21]. In this study, higher anisometropia was associated with a higher accommodation lag. Hyperopic defocus associated with accommodation lag leads to an increase in the axis; therefore, we speculate that higher accommodation lag is one of the factors contributing to myopia progression in patients with anisometropia. However, a previous study found that increased lag in accommodation occurs after the onset of myopia in children [22]. This result indicates that increased hyperopic defocus owing to accommodation may be a consequence rather than a cause of myopia.

Previous research found that children with myopia have a higher AC/A ratio than those with emmetropia. The AC/A ratio rises 4 years before the onset of myopia and remains stable and elevated for a minimum of 5 years after the onset [23]. This may be owing to the higher gain of the cross-link from accommodation to convergence, or it may represent an increase in the desired conditioning output per diopter, although the accommodative convergence cross-link gain relationship may be relatively constant. The literature on AC/A in anisometropia children is limited. In our study, the gradient AC/A ratio decreased significantly with the increase in anisometropia. This reflects the imbalance between binocular accommodation and convergence in patients with anisometropia. This result may be owing to unequal accommodation demands for both eyes. When individuals look at an object from a distant point, it tends to use the eye with less accommodation, while the other eye is nonaccommodating [24]. Therefore, when patients with myopic anisometropia look at objects, the accommodation ability, adaptive convergence linkage ability, and AC/A ratio decrease. This study is inconsistent with the results of the above increased AC/A in children with myopia compared with the normal group. This contradictory result may be owing to the following two reasons. Firstly, the previous studies were not conducted in patients with myopic anisometropia and were inconsistent in the age range. Secondly, the mechanism of accommodation of imbalance between eyes in children with anisometropia differs from the gain of the cross-link from accommodation to convergence in those with myopia.

Stereopsis is the ability to perceive the visual space depth through the sensory fusion of the retina of both eyes in the third dimension. Our study found that anisometropia is positively correlated with near stereopsis, and the higher the anisometropia, the more obvious the damage of near stereopsis. The range of normal stereopsis is 40–60 s of arc. A study of 2376 children aged 7–14 years demonstrated that the stereopsis proportion below normal was 6.82% [25], which was comparable to 5.4% to 12.3% proportions in previous studies [26, 27]. This suggests that abnormal stereopsis accounts for a large proportion. The eye with higher myopia typically results in reduced visual acuity and defocused retinal images in patients with anisometropia. This leads to asymmetric signals from both eyes and insufficient neuronal development at the brain level [28]. Therefore, correction must be performed as early as possible in the early stage of children's visual development to prevent increased refractive error, binocular visual impairment, and even amblyopia.

## 5. Conclusion

This study sets out to explore the relationship between anisometropia and binocular vision in children with myopic anisometropia. The study demonstrated that binocular visual impairment develops with the increase in the interocular difference. The binocular visual function should be fully considered before correction for children with myopic anisometropia. This study's findings may provide a deeper understanding of the relationship between binocular visual function and anisometropia in children with myopia for reality clinical work.

## Figures and Tables

**Table 1:** Comparison of demographics among three groups.

	Isometropia	Low anisometropia	High anisometropia	*F*/*χ*^*2*^	*P*
*n*	35	42	33		
M/F	15/20	20/22	14/19	0.261	0.878
Age (y)	10.66 ± 1.89	11.69 ± 2.21	11.79 ± 2.59	2.764	0.068
Time of myopia (y)	1.26 ± 1.44	1.39 ± 1.76	3.79 ± 2.59	18.115	<0.001
SER (D)	0.37 ± 0.31	1.61 ± 0.40	3.28 ± 0.77	267.665	<0.001

M: male; F: female; SER: spherical equivalent refraction; Chi-square test was used for sex, and univariate ANOVA was used for age, time of myopia, and SER.

**Table 2:** Comparison of accommodation and binocular vison parameters among three groups.

Parameters	Isometropia	Low anisometropia	High anisometropia	*F*/*H*	*P*
Distance heterophoria (^∆^)	−1.00 (−2.25, 0.50)	−1.50 (−3.50, −0.50)	−3.00 (−6.00, −1.00)^#^	13.722	0.001
Near heterophoria (^∆^)	−2.79 ± 5.88	−3.87 ± 5.88	−6.76 ± 7.17^#^^*∗*^	3.683	0.028
NRA (D)	2.5 (2.25, 2.75)	2.5 (2.0, 2.75)	2.75 (2.5, 2.75)	3.197	0.202
PRA (D)	−3 (−3.75, −2.50)	−2.875 (−4.25, −1.75)	−2.25 (−3, −1.75)^#^	6.636	0.036
AR (D)	0 (0, 0.25)	0 (0, 0.25)	0.25 (0, 0.5)^#^^*∗*^	10.45	0.005
AMP (D)	16 (13.5, 16.5)	13.75 (11.1, 16)^#^	13.5 (12.5, 14.6)^#^	14.246	0.001
Average age-expected AMP (D)	15.2 (15.2, 15.8)	15.05 (14.6, 15.5)	14.9 (14, 15.5)	5.11	0.078
Differences (D)	0.2 (−2, 1.15)	−1.15 (−4.1, 0.7)^#^	−1.5 (−2.3, −0.8)^#^	11.811	0.003
AC/A ratio (^∆^/D)	4 (3, 5)	4 (3, 5)	3 (2, 4)^#^^*∗*^	9.717	0.008
Near stereopsis (log)	1.51 (1.30, 1.65)	1.70 (1.40, 2.00)^#^	1.80 (1.80, 2.20)^#^^*∗*^	30.661	<0.001

NRA: negative relative accommodation; PRA: positive relative accommodation; AR: accommodative response; AMP: amplitude of accommodation; the average age-related AMP: 18.5 − 0.3 *∗* age; Differences: AMP measured value minus average; AC/A: accommodative convergence over accommodation; univariate ANOVA was used for near heterophoria, and the Kruskal–Wallis *H* was for other values; compared with the control group, ^#^*P* < 0.05; compared with the low anisometropia group, ^*∗*^*P* < 0.05.

**Table 3:** Correlations of anisometropia and binocular vision parameters.

IOD (D)	*r*	*P*
Age	0.304	0.001
Distance heterophoria	−0.259	0.006
Near heterophoria	−0.201	0.036
AR	0.306	0.001
AMP	−0.315	0.001
Near stereopsis	0.535	<0.001

IOD: interocular difference; AR: accommodative response; AMP: amplitude of accommodation.

## Data Availability

The data used in this study are available upon reasonable request.
